# Case of Morbus Cordis,—Ossification of Sigmoid Valves

**Published:** 1845-10-01

**Authors:** 


### Case of Morbus Cordis; Ossification of Sigmoid Valves.

Communicated for the Buffalo Medical Journal, by the EDITOR.

The following case is chiefly interesting as affording an illustration of the validity of physical signs in the diagnosis of valvular disease, and the practicability of detecting the particular valves affected. The object in reporting it, is to incite attention to the principles of diagnosis in cardiac affections as developed by the researches of modern pathologists in this department, and which are most lucidly and admirably presented to the student in the last edition of Hope's treatise, with notes by Dr. Pennock of Philadelphia, recently published in this country. To avoid extending the length of the article, the report will be confined to the condition of the heart as determined before death by the signs and symptoms, and its morbid anatomy as revealed by dissection.

The following is an account of an examination of the case transcribed from my case-book, which was recorded Feb. 6th, 1845: "I examined to-day J. M., aged 70 years, whom I had suspected from his symptoms, of having valvular ossification. He has been subject to cough, with occasionally free, serous expectoration, for a great number of years; has a little oedema of feet at times, not constantly; never suffers from palpitation; without any difficulty of respiration; can lie with his head low—the pulmonary symptoms, in short, alone indicate pathological effects directly resulting from cardiac obstruction. On a general examination by percussion, the dulness extends over a larger space than natural; but I did not examine with precision with reference to this, nor did I ascertain whether the point of impulsion against the chest was normal, it not being convenient to denude the chest sufficiently to ascertain these points. The force of impulsion against the chest is not very decidedly increased. Pulse did not present marked force, nor was it jerking. It would follow from these circumstances, that hypertrophy does not exist to a great degree, nor is there great obstruction at the orifices. On application of the stethoscope on the left margin of the sternum as high as the 3d rib, the *bruit de soufflet*, or bellows murmur was very audible: it could be heard with scarcely diminished intensity as high upward as the clavicles. Below the location of the semilunar valves it obviously, and rapidly diminished in intensity. At the apex of the heart it could not be heard so loudly as above the location of the sigmoid valves. According to my present recollection, it was of a loud pitch; probably as high as R whispered. Its quality was that of roughness—not a soft, puffing murmur as in the case following, [in note book.] What is the diagnosis? Ossification, especially of the sigmoid valves, where the audible murmur is chiefly produced, without great hypertrophy, or obstruction, or regurgitation. It is interesting that in this case, in which there is obviously valvular disease, there are so few, and, indeed, no distressing symptoms referred by the patient to the heart—no pain, dyspnoea, palpitations, &c. It illustrates the fact which Hope states, that osssification, uncomplicated with much obstruction, or hypertrophy, is not a distressing affection, and not incompatible with long life.

On May 17th the following note was appended to the preceding record: "Examined yesterday and verified above. The pitch of the murmur is not so high as R, nor so low as *who*. There is no second sound in this case."

The patient died in the latter part of July following. He lapsed slowly into a state of stupor, and, at length, coma, with great prostration, aggravated by frequent paroxysms of diarrhœa. He seemed to succumb from old age, and general exhaustion from the diarrhœa.

A partial dissection was obtained—sufficient to determine the condition of the heart, and the examination was chiefly limited to this object. The examination was held 36 hours after death, when the body had become quite offensive, the weather being extremely warm. Present at the examination, Drs. F. H. Hamilton, and J. P. White.

The heart, without being measured, was estimated to be moderately hypertrophed, with dilatation of its cavities. Its texture was softened, but it was difficult to say how much of this was due to cadaveric decomposition. The aorta, just above the sigmoid valves, was dilated—say about one third, or one fourth greater than its natural calibre. Several ossific points were discovered on the lining membrane of the aorta, and a single scale of bone 4 or 5 lines in length, and 2 or 3 in breadth. At the base of two of the sigmoid valves, ossific matter was present, forming an apparent aggregation of small, distinct depositions. The valves themselves, however, were not so much affected as to prevent their expanding and collapsing, so as to fulfil, measurably, their functions. The third was permanently expanded, and rigid, considerable ossific matter being deposited at its base. The mitral valves were sound, with exception that the chordæ tendinæ appeared more rigid than usual, and a single filament had become ossified. Their natural play, however, could not have been materially disturbed.

The right sigmoid and auriculo-ventricular valves were healthy.—The lungs, on a superficial examination, appeared sound. The liver presented numerous collections of a yellow fluid, resembling bile, commingled with partially coagulated blood. These accumulations were from the size of a pea to that of a filbert. The biliary vessels seemed to be congested with bile, which exuded freely from divided surfaces. It was not very minutely examined, and the dissection was not extended to other organs. *Buffalo, September*, 1845.

